# Impulse dispersion of aerosols during playing wind instruments

**DOI:** 10.1371/journal.pone.0262994

**Published:** 2022-03-03

**Authors:** Sophia Gantner, Matthias Echternach, Reinhard Veltrup, Caroline Westphalen, Marie Christine Köberlein, Liudmila Kuranova, Gregor Peters, Bernhard Jakubaß, Tobias Benthaus, Michael Döllinger, Stefan Kniesburges

**Affiliations:** 1 Division of Phoniatrics and Pediatric Audiology, Department of Otorhinolaryngology, University Hospital, LMU Munich, Munich, Germany; 2 Division of Phoniatrics and Pediatric Audiology, Department of Otorhinolaryngology, Head & Neck Surgery, University Hospital Erlangen, Medical School, Friedrich-Alexander-University Erlangen-Nueremberg, Erlangen, Germany; 3 Institute and Clinic for Occupational, Social and Environmental Medicine, University Hospital, LMU Munich, Munich, Germany; Public Health England, UNITED KINGDOM

## Abstract

Musical activities, especially singing and playing wind instruments, have been singled out as potentially high-risk activities for the transmission of SARS CoV-2, due to a higher rate of aerosol production and emission. Playing wind instruments can produce condensation, droplets of saliva, and aerosol particles, which hover and spread in the environmental air’s convectional flows and which can be potentially infectious. The aim of this study is to investigate the primary impulse dispersion of aerosols that takes place during the playing of different wind instruments as compared to breathing and to speaking. Nine professional musicians (3 trumpeters, 3 flautists and 3 clarinetists) from the Bavarian Symphony Orchestra performed the main theme from the 4^th^ movement of Ludwig van Beethoven‘s 9^th^ symphony in different pitches and loudness. The inhaled air volume was marked with small aerosol particles produced using a commercial e-cigarette. The expelled aerosol cloud was recorded by cameras from different perspectives. Afterwards, the dimensions and dynamics of the aerosol cloud were measured by segmenting the video footage at every time point. Overall, the flutes produced the largest dispersion at the end of the task, reaching maximum forward distances of 1.88 m. An expulsion of aerosol was observed in different directions: upwards and downwards at the mouthpiece, at the end of the instrument, and along the flute at the key plane. In comparison, the maximum impulse dispersions generated by the trumpets and clarinets were lower in frontal and lateral direction (1.2 m and 1.0 m towards the front, respectively). Also, the expulsion to the sides was lower.

## Introduction

In order to limit the person-to-person transmission of COVID-19, gatherings of people, commonplace for in cultural institutions and music events, have been restricted worldwide. Transmission of COVID-19 is suspected to happen by direct contact, by small saliva droplets with diameters ≥5 μm, and by aerosols with diameters ≤ 5 μm [1]. As some outbreaks of the disease were associated with choir rehearsals or concerts [2–4], these events have been singled out as potentially high-risk activities for the transmission of SARS CoV-2, which was confirmed by the fact that singing is associated with a much higher rate of aerosol emission than breathing and speaking [5, 6].

Similarly, playing wind instruments can produce aerosols, droplets of saliva and condensation water, which can be potentially infectious if the musician is infected with the SARS CoV-2 virus, also if the musician is asymptomatic. Whereas the droplets in general fall to the ground after a certain distance, the aerosols remain hovering in the air, owing to their small mass, and convectively spread in the environment. Consequently, infections appear possible due to exposure or inhalation of virus containing aerosols in closed rooms. The majority of aerosol particles are below one micron, so we will refer to them as sub-micron particles.

Particularly, straight, long instruments with conically increasing diameter like vuvuzelas have the capacity to propel large numbers of aerosols into the air which are able to penetrate as far as the lower lung of another person [7]. In contrast, a few studies measuring the concentration and the size of airborne particles from brass and woodwind instruments reported low emission rates at distances of .5 m to 4 m [8]. He et al. categorized 15 different wind instruments into low (tuba), intermediate (bassoon, piccolo, flute, bass clarinet, French horn and clarinet) and high risk (trumpet, bass trombone and oboe) levels based on a comparison of their aerosol generation with those from normal breathing and speaking [9]. The aerosol concentration and outlet flow and varies with changes in music amplitude, pitch, and note duration, with different relationships were observed for each instrument depending on geometry and playing technique [10].

Previous studies measured the air velocity and movement emerging from wind instruments using photography, Schlieren optics, sensors in the vicinity of the musician and Particle Image Velocimetry [11–15]. The latter observed an airflow up to .5 m from the bell of brass instruments like trumpet, trombone and euphonium. In woodwinds, the flute, particularly, caused an airflow over a distance of more than 1 m [14]. In a previous study by our group [16], artificially-added aerosols were analysed with respect to the impulse dispersion characteristics in professional singers for singing and speaking tasks. It was found that the maximum impulse dispersion to the front was up to 1.4 m for singing and less pronounced to the sides. For wind instruments, however, the temporal and spatial spreading characteristics of aerosols have not yet been investigated. The aim of this study is to reveal the aerosol expulsion characteristics of wind instruments. The intention was to show the spreading of the expelled potentially virus-laden aerosol particles immediately during playing.

## Material and methods

With approval from the local ethical committee (Ethikkommission der Medizinischen Fakultät der LMU München: 20–395), 9 professional musicians (1 female, 8 male, age 48±4 years) from the Bavarian Symphony Orchestra (Symphonieorchester des Bayerischen Rundfunks) were included in the study. Informed written consent was obtained from all the participants. All of them were non-smokers without pulmonary symptoms. Three of them were trumpeters, three flautists and three clarinetists. All participants practice western classical music and had completed their instrumental studies at a University of Music. None of them suffered from dysphonia at the time of the study: The values for the Voice Handicap Index 12 (VHI-12) in a German version [17] exhibited norm values (mean 3 SD4).

From all participants shown in the figures, informed written consent to publish the images in an online open-access publication was obtained. All methods were carried out in accordance with relevant guidelines and regulations.

### Tasks

All participants performed a part of the main theme (melody: M) of the fourth movement of Ludwig van Beethoven‘s 9^th^ symphony in the key of D major. The musicians played the melody in their specific ranges of “high” (h) and “low” pitch (l). Additionally, they were asked to play the high pitch task in a loud (Mh+) and a soft version (Mh-). To sum up, there were three different tasks for each instrument: melody on a high pith and loud (Mh+), melody on a high pith and soft (Mh-) and melody on a low pitch and loud (Ml+). Focused on the worst conditions regarding sub-micron particles dispersion during playing wind instruments, the largest expansion was expected for Mh+. Based on this, the influence of a low pitch (Ml+) and low sound level (Mh-), yielding a smaller expansion for both, was investigated. A task Ml- which combines both effects and permits the expectation of much smaller expansion distances, was not investigated.

The specific ranges for each instrument are as follows: (Mh: trumpet: absolute pitch range: D5—A5, fundamental frequency (ƒ_o_) range: 587–880 Hz; Mh: flute: D6—A6, 1175 – 1750 Hz; Mh: clarinet: Bb4—F5, 466 - 699 Hz); (Ml: trumpet: D4—A4, 294 - 440 Hz; Ml: flute: D5—A5, 587 - 880 Hz; Ml: clarinet: Bb3—F4, 233 - 349 Hz).

In addition, there were specific tasks for each instrument:

- The trumpeters performed two further tasks, playing with the mouthpiece only and without the mouthpiece by just buzzing the melody with the lips.
- The clarinetists played the melody with mouthpiece only. Additionally, they produced their lowest pitch (147 Hz), where all keys and holes are closed
- The flautists performed the task using the so-called fluttering tongue technique.

All these tasks lasted approx. 6 s, corresponding to a speed of 80–90 bpm for the M tasks.

In addition to the instrumental tasks, each musician spoke the affiliated original text (T) („Freude, schöner Götterfunken, Tochter aus Elysium“) from Friedrich Schillers poem “An die Freude” at a comfortable pitch and average loudness.

In a pre-experiment the Forced Expiratory Pressure in 1 Second (FEV1) and Tiffeneau-Index (TIFF Index = FEV1/VC) were measured in order to investigate the pulmonary function with a spirometer (ZAN100, Oberthulba, Germany).

### Test setup

**Fig 1:**
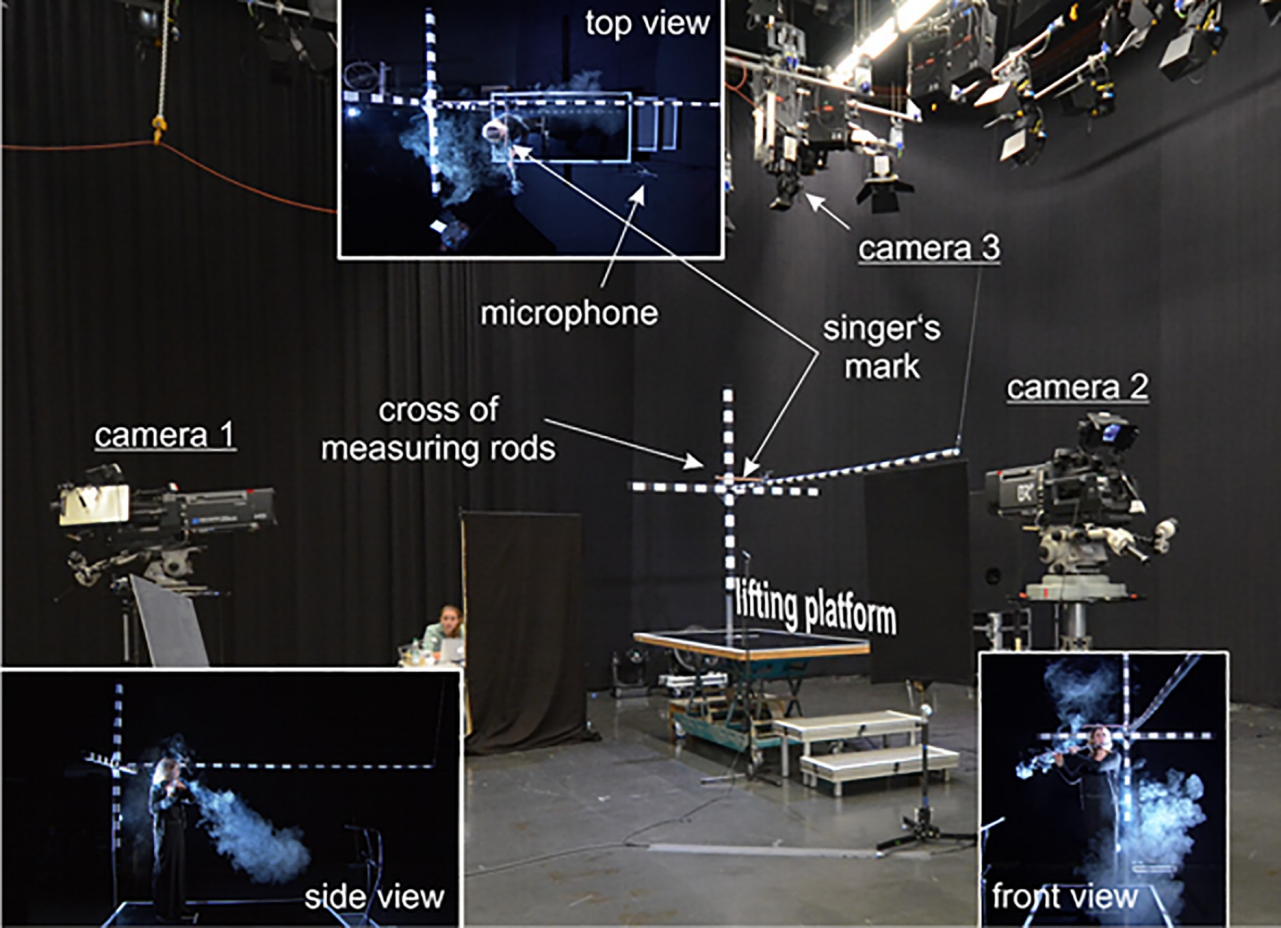
Picture of the test setup including the three cameras, the lifting platform and measuring rod cross (spatial directions are indicated by x, y and z). The perspective views of each camera are shown with a musician in performing position.

For the experiments, all subjects inhaled the smoke of an e-cigarette that had been filled only with the basic liquid, which consists of 50% glycerin and 50% propylene glycol. A Lynden Vox e-cigarette was used to nebulize this liquid, (Lynden GmbH, Berlin, Germany). The particles generated in e-cigarettes have a diameter in the range of 250 - 450 nm and are thus in the range of aerosols expelled during breathing and talking [16, 18]. For each task, a spirometer (ZAN100, Oberthulba, Germany) was coupled with the mouthpiece of the e-cigarette to measure the individual inhaling volume. Each musician stood on a lifting platform to raise them to the correct height. At the level of the forehead, a mark was placed to show the musician’s correct position on the platform, as shown in the top view part of Fig 1. While standing on the platform, each musician inhaled a similar volume of smoke with an average of 0.76 litres (SD 0.26 litres) of smoke, controlled by the spirometer. Immediately after inhalation, each musician moved to the mark and started to perform the task. After they had completed the task, the musicians were instructed to maintain their position on the platform without moving for 60 s with the aim of tracing the exhaled cloud. Because all musicians were non-smokers, they were instructed how to inhale correctly prior to the actual experiments. In cases of sudden coughing, the task was repeated until it could be performed correctly. The e-cigarette smoke changed some subjects’ self-sensation during playing–presumably due to changes of the resonance properties of the instrument and the gas column. The professionally trained subjects could, nevertheless, compensate for these changes during the experiment, applying their playing technique also under the experimental circumstances.

All measurements were conducted in a studio with the dimension of approximately 27 m x 22 m x 9 m (width x length x height). The setup is depicted in Fig 1. The distance to the wall behind the platform was 4 m and 5 m to the left. The distance between the platform and the frontal and the right wall was more than 7 m. Three measuring rods for each spatial direction were mounted at the lifting platform to enable conversion of the pixel dimensions of the recorded picture into metric dimensions. The rods were assembled in a cross configuration and had a 2 cm and 10 cm scale, respectively.

**Fig 2:**
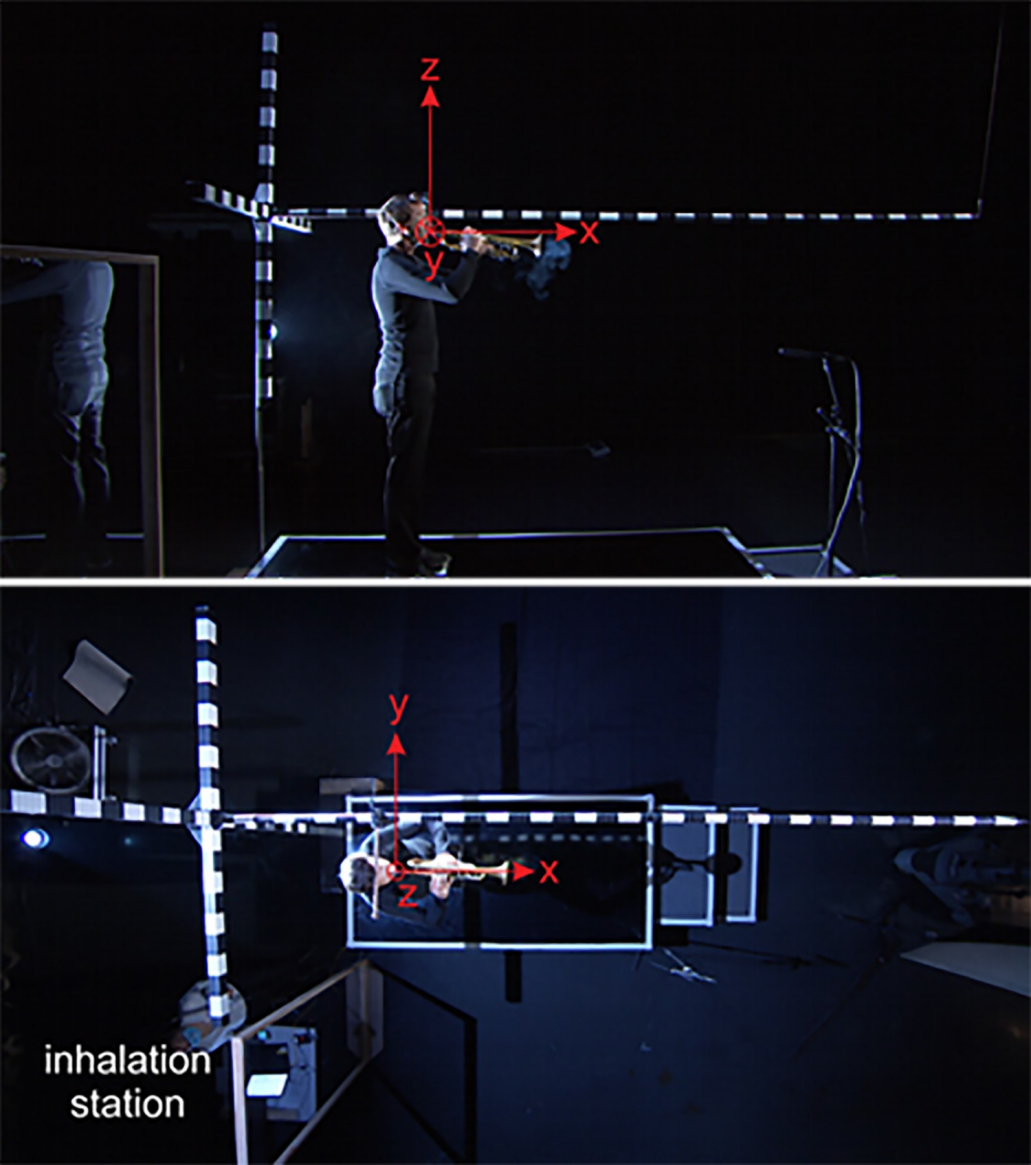
Pictures of a trumpeter in the side view (upper) and top view (lower) perspective. The origin of the coordinate system was placed at the mouth opening of the instrument player.

Three full HD Sony HDC 1700R cameras of the Bavarian Broadcast (resolution 1920 x 1080 pixels) recorded the musicians and the cloud from side, top, and front view perspectives, as shown in Fig 2. The side and front cameras were equipped with Canon DIGI SUPER 25 XS lenses (Canon, Tokio, Japan) and the top camera with a HD Fujinon HA14x4.5BERM/BERD wide angle HD lens (Fujinon, Tokio, Japan). All cameras recorded synchronously at a rate of 25 fps.

The audio signal was recorded with a Sennheiser KMR 81 directional and a ME62 omnidirectional microphone (Sennheiser electronic GmbH, Wedemark, Germany) which was placed at the lower right corner of the lifting platform, at a distance of approx. 1.5 m to the musician’s mouth, as shown in the top view zoom in Fig 1. For estimation of the sound pressure level (SPL), the microphones were calibrated with the Sopran software (Svante Granqvist, Karolinska Institut, Stockholm, Sweden) using a sound level meter (Voltcraft, Hong Kong, China).

In order to achieve a high contrast between the cloud of smoke and the background, the entire studio was covered with black linen and the musicians wore black clothes. The smoke was illuminated with three spotlights positioned on the left hand side of the platform: one in the left corner behind the platform with a distance of approx. 5 m to the musician, one in front of the platform on the left hand side at a distance of 7 m and another directly behind the platform approx. 3.5 m from the rear edge of the platform.

Before each task, the studio was aerated with the main gate open for at least 2 min using a fan behind the platform. The main gate was at the opposite side of the studio with a distance of approx. 20 m to the platform. Afterwards, the gate was closed and all participants were instructed to stop moving for another 2 min to let air circulation settle down. Subsequently, the task started with the musician’s inhalation of the e-cigarette smoke. The temperature and the humidity in the studio were measured. The mean temperature was 23°C (SD .46) and the relative humidity 46% (SD .95).

### Segmentation analysis

The expelled vapor cloud was segmented in the footage of the side and top view cameras by an in-house software tool, yielding its contour as a function of time in order to track its temporal evolution, so the dispersion of the air which is potentially laden with sub-micron particles was measured and marked. Therefore, (1) the side and top camera recordings were converted into gray scales and (2) the subjects and bright equipment parts in the region of interest (i.e. parts of the subjects’ skin of their hands, the white sectors of the measuring rods) were covered with black masks using the software Sensarea (Grenoble Institute of Technology (INPG), France). A coordinate system was defined with its origin at the subjects’ mouth, see Fig 2. Then, the cloud contour was segmented in each frame of the video, using a threshold-based region-growing algorithm.

After the segmentation, the maximum expansions of the cloud in each frame were identified in all three spatial directions (x, y, z). In the final results, showing the maximum cloud expansion from the mouth, several outliers occurred if non-smoke regions with a similar grayscale were segmented when the cloud came close. Those outliers were removed in two steps: (1) a moving median filter with a fixed window length of 30 time points and (2) a subsequent cubic spline fitting approximation. The computation of curve smoothing was performed in Matlab (The Mathworks Inc., Natick, MA).

## Results

In the pre-experiment (spirometry only) without inhalation of the basic e-cigarette smoke, the subjects showed lung function parameters with a median FVCin of 4.53 litres (SD = 0.66 litres) and a TIFF Index of 86 (SD 4.3). The volume of smoke of basic liquid inhaled with an e-cigarette immediately before each task was on average 0.76 litres (SD 0.26 litres).

All subjects completed the tasks without major problems, even though the sound generation properties of the instruments changed by the higher density of the smoke in comparison to air. No subject had to be excluded. For the breathing task, a median distance over all subjects of 1.01 m (SD .51 m) in front (x), .36 m (SD .24 m) in vertical (z) and .21 m (SD .32 m) in horizontal, transversal direction (y) were detected. The median distance for speaking amounts .54 m (SD .24 m) to the front (x), .37 m (SD .17 m) in vertical (z) and, .30 m (SD .10 m) in horizontal transversal direction (y). During speaking, the exhaled air has a lower velocity than during breathing due to the higher flow rate for aspiratory air exchange in the lungs. Thus, the smoke travelled over a smaller distance compared to breathing. These data were used for comparison with the following measurements during instrumental playing.

For the evaluation of the playing tasks Mh+, Mh- and Ml+, the distances were taken at the end of the task which represents the time point 0 in Figs 4–6.

### Trumpets

Fig 2 shows the representative images in different camera views while the Mh+ task is performed by a male subject.

The median distances of the aerosol impulse dispersion generated from the three trumpeters during the playing tasks Mh+, Mh- and Ml+ amount .86 m in x-, .30 m in y- and .02 m in z-direction at the end of the task. The maximum distances were 1.2 m in x-, .52 m in y- and .52 m in z-direction.

The time evolution of the aerosol cloud is shown in Fig 4 for each player and the tasks Mh+, Mh- and Ml+. In all spatial directions, the main distance was generated before the end of task; i. e., at time point t = 0 s. In the further progress, the motion of the cloud decelerated and reached a constant distance at t = 15 s. In some cases, the distance became smaller after the task’s end which was caused by the dilution of the cloud, by the hovering of the cloud or by exceeding the region of interest of the measurement. In those cases, the cloud vanished and could not be segmented any more.

The curves representing the distance in x, start at x = .5 m, Fig 4A). The reason is that the origin of the coordinate system is placed at the player’s mouth, but the cloud predominantly exited at the bell of the trumpet, approx. 50 cm further downstream in x-direction.

In the y-direction, the cloud tended to move in positive direction even after the task had ended. This tendency can also be seen in Figs 5B and 6B for the flutes and clarinets. In accordance to the study with the singers [16], it could be assumed that this cloud motion was produced by the motion of the player from the rear end of the platform where they inhaled the sub-micron particles to the marked performance spot.

Regarding the vertical direction, the cloud tended to move in a positive z-direction. The tasks of playing with the mouthpiece only and with buzzing reached similar distances of .86 m and .9 m in x-direction, respectively. The expansion in y-direction was found to be slightly larger with .68 m and .47 m. The expansion in z-direction was .11 m and .09 m.

### Flutes

**Fig 3:**
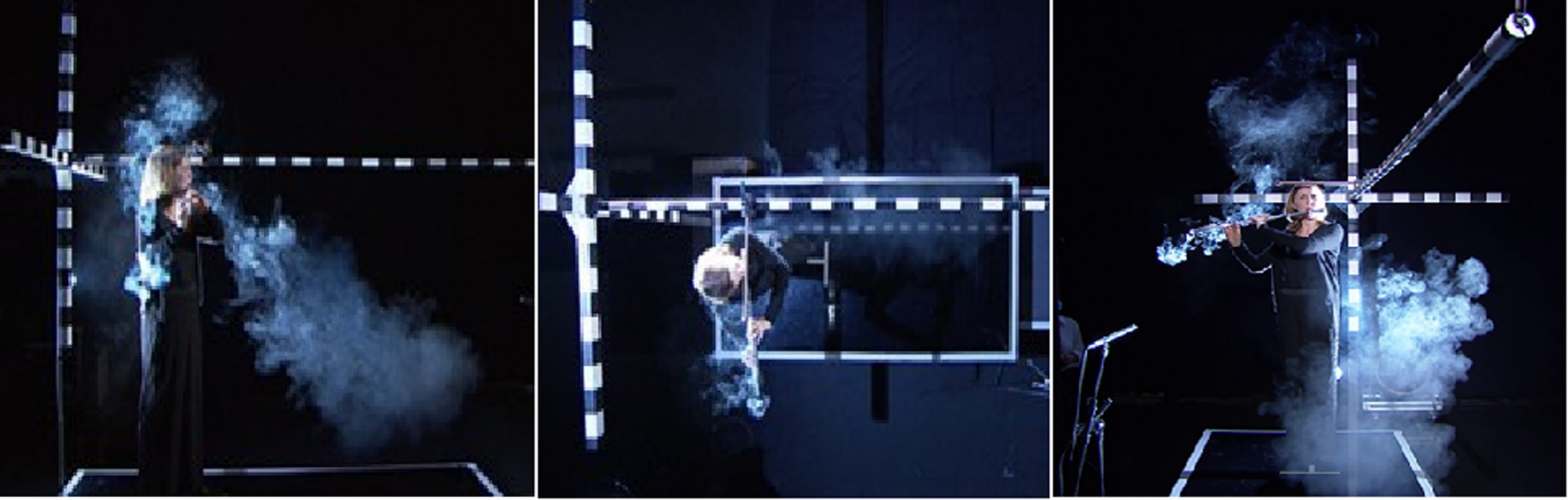
Pictures of a flute player (from left to right) in the side view, top view and front view at the test setup while playing the melody. Three clouds were developed from the flute: at the mouthpiece, at the end of the instrument and from the key plane.

In the case of the flutes, an expulsion of sub-micron particles in different directions has been observed: upwards and downwards at the mouthpiece, at the end of the instrument, and along the flute at the key plane, as shown in Fig 3. Overall, the flutes produced the largest median distance at the end of task of 1.51 m in the front (x), .64 m in lateral direction (y) and .02 m in vertical direction (z) considering all three playing tasks and players. The maximum distance was 1.88 m in x-, 1.29 m in y- and 1.12 m in z-direction (Fig 5).

According to Fig 3, the large distances of the aerosol motion in x-direction was achieved by the expulsion at the mouthpiece owing to the high velocity of the player’s expiration flow. The sub-micron particles ejection at the key plane and the end of the flute occurred with a much smaller velocity.

Regarding the temporal evolution of the aerosol cloud spreading, the main distance was again generated in the period before the end of task (t = 0 s). Afterwards, the cloud decelerated to stagnate at a constant distance or even dissolved, which was represented by a decrease in distance. The motion trend in a positive y-direction can also be seen but is not as distinct as for the trumpets and clarinets.

In an additional experiment, the flutists played in flutter-tongue style. This resulted in similar median distances of 1.56 m in x-, .40 m in y- and .56 m in z-direction, compared with the normal playing style.

### Clarinets

The clarinets showed sub-micron particles ejections at the mouthpiece, at the end of the instrument (main outlet) and at the key plane. The median distances of all tasks and players were measured to be 1.0 m in x-, .55 m in y- and .51 m in z-direction. The maximum distance in x-direction reached by a clarinet player was 1.51 m, 0.95 m in y- and 1.1 m in z-direction.

Fig 6 shows the temporal evolution of the aerosol expansion during playing the clarinets: The main distance is reached before the end of task with a subsequent deceleration, resulting in a constant distance or decay of the cloud, leading to a decrease of distance, predominantly for the x-direction. The motion tendency in positive y-direction is again clearly visible, as shown in Fig 6B).

For the additional tasks of playing with mouthpiece only, a median distance of .90 m in x-, .86 m in y- and .80 m in z-direction was observed. Furthermore, the cloud was examined for playing the lowest pitch possible, with all keys kept closed. As a result, the entire air flow exited via the main outlet at the end of the instrument. Here, similar values, compared to the main tasks with median distances of .96 m (x), .17 m (y) and - .18 m (z) were detected.

## Discussion

**Fig 4:**
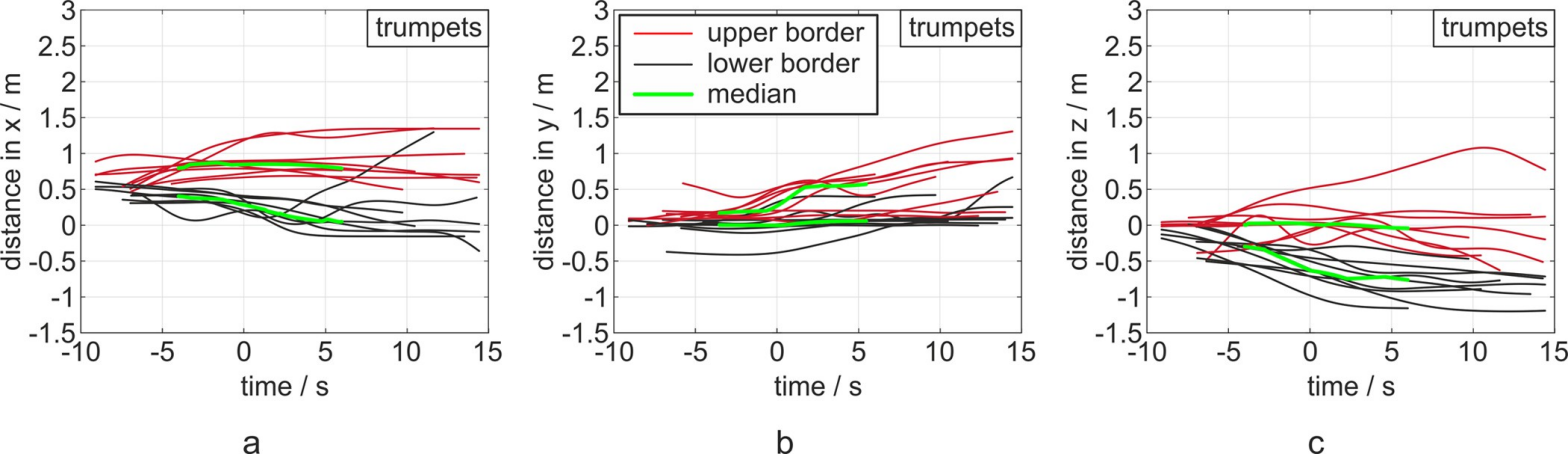
Diagrams of distances in x-, y- and z-direction (a, b, c) from left to right for the trumpets. The 0 point in the time-scale refers to the end of the task. Each task is represented by a red (upper border) and a black curve (lower border) indicating the maximum distances of the cloud in the respective directions. The median devolution in both directions is shown in green. The corresponding diagrams with color-coded notation of the three tasks are displayed in the S1 Fig.

Musical activities especially singing and playing instruments, are assumed to contribute to the transmission of CoVID19 by aerosols [19–21]. The presented data showed that the expulsion and spreading of sub-micron particles during playing the flute reached, in particular cases, in a short amount of time, distances to the front of up to 1.88 m, for trumpets up to 1.2 m and for clarinets up to 1.2 m. For one of the clarinet players, the cloud spread up to 1.51 m, as shown in Fig 4A, but this can be explained by a convectional flow to the left and front, as explained below. Furthermore, the data reveal that the distance of dispersion is similar for loud and soft playing, as well as playing only with mouthpiece. In contrast, speaking showed much lower dispersions.

In order to protect other people from potentially infectious particles during the CoVID19 pandemic, it seems important to understand the temporal and spatial dispersion dynamics for sub-micron particles, considering the region of the immediately emitted aerosol cloud. The study focused on the dispersion of the exhaled flow due to the initial momentum. After the musicians stopped playing, the sub-micron particles certainly continue to spread in the room across large distances which is, however, driven by secondary flows in the room (e.g. ventilation, thermic conditions, movements of persons, …). These influences are highly situation-dependent regarding the local conditions and were not investigated in the study. The presented data showed that for all instruments the impulse dispersion to the front was below 1.5 m at the end of the task. The only exception was the flute reaching distances up to 1.88 m.

The transmission risk during the pandemic is not only dependent on the impulse characteristics analysed in the presented study, but also on the absolute aerosol generation during playing. He et al. [9] measured the absolute aerosol generation and estimated that the trumpet (among bass trombone, and oboe) is among the instruments with a high sub-micron particles emission rate. The aerosol production has been assumed to be caused by the lip oscillation during trumpet playing. However, in the presented study, only a small aerosol cloud was emitted. We assume, that a large part of the aerosol cloud remained in the trumpet and a smaller fraction exited the instrument. Furthermore, in agreement with the observation by He et al., the impulse of the exiting cloud was low, showing only a small velocity and so a small dispersion of the aerosol cloud to the front. Moreover, it remains unclarified to what extent the aerosols remain inside the trumpet as particles or condensed liquid. In a single subject test, a cleaning flow 2 min after the experiment still showed an ejection of the remained vapour. In contrast to the trumpets, the absolute aerosol concentrations of the flute and the clarinet measured by He et al. [9] was assigned to an intermediate risk level compared with aerosol generation during speaking and breathing. The impulse of the ejected sub-micron particles, however, was largest for the flute, generating a very high velocity. Regarding the estimation of a transmission risk due to instrument playing, the results of both studies have to be evaluated in conjunction: He et la. Showed the amount of produced and ejected sub-micron particles, and this study shows how far these particles are expelled around the instrument player.

The large variability of sub-micron particles expulsion and spreading is attributed to combined effects of different mechanisms of sound production for instrument types, mouthpiece and tube structure. One representative instrument each for brass, woodwind and flute was selected for the study. Among the brass instruments, the trumpet has the smallest bell size and correspondingly the highest outlet velocity, the tube structure is closed and the design is, comparable to trombone or horn, straight forward. It can be assumed that the measurement for trumpets will cause less artefacts, as they could occur due to the movements a trombone tension rod or the curved design of a horn. The flute generates three aerosol clouds due to the airflow refraction at the mouthpiece, the keys and the opening at the end. The clarinet was chosen as a representative of the reed instruments, because a larger cloud was expected in comparison to the oboe due to the construction and the blowing technique.

It is questionable to what extent the results of the investigated instruments can be transferred to instruments of similar construction. In this context, the results from the clarinets could be transferred to other reed instruments, such as the oboe or the English horn. Similarly, the results of the trumpet could help to estimate the aerosol dispersion of the tuba, the trombone or the horn. However, in the case of double-reed instruments like the oboe or the English horn, there seems to be a much tighter seal around the mouthpiece, so that less sub-micron particles might be emitted. The dispersion from the main outlet can be assumed to be similar, as the straight tube structure is comparable. The mouthpiece of the trombone, horn or tuba is similar to that of the trumpet, so that a similarly tight seal with the lips may be ensured and therefore hardly any sub-micron particles would escape. It is conceivable that due to the longer and more convoluted tube system of the above instruments, sub-micron particles are deposited and thus less aerosol is emitted by the main outlet.

**Fig 5:**
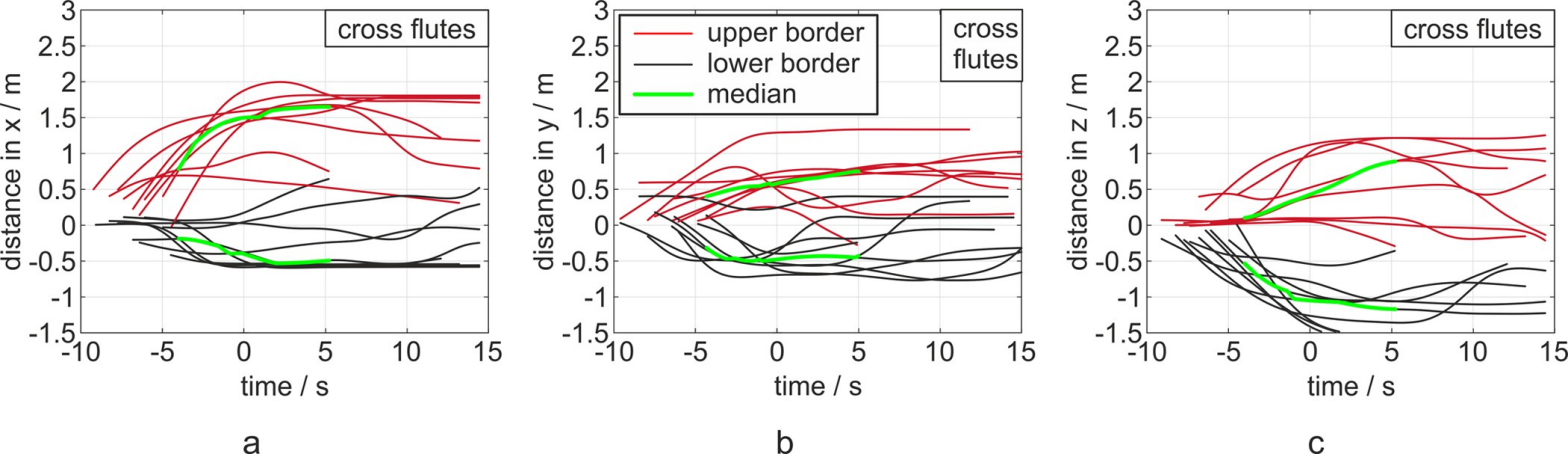
Diagrams of distances in x-, y- and z-direction (a, b, c) from left to right for the flutes. The 0 point in the time-scale refers to the end of the task. Each task is represented by a red (upper border) and a black curve (lower border) indicating the maximum distances of the cloud in the respective directions. The median devolution in both directions is shown in green. The corresponding diagrams with color-coded notation of the three tasks are displayed in the S2 Fig.

**Fig 6:**
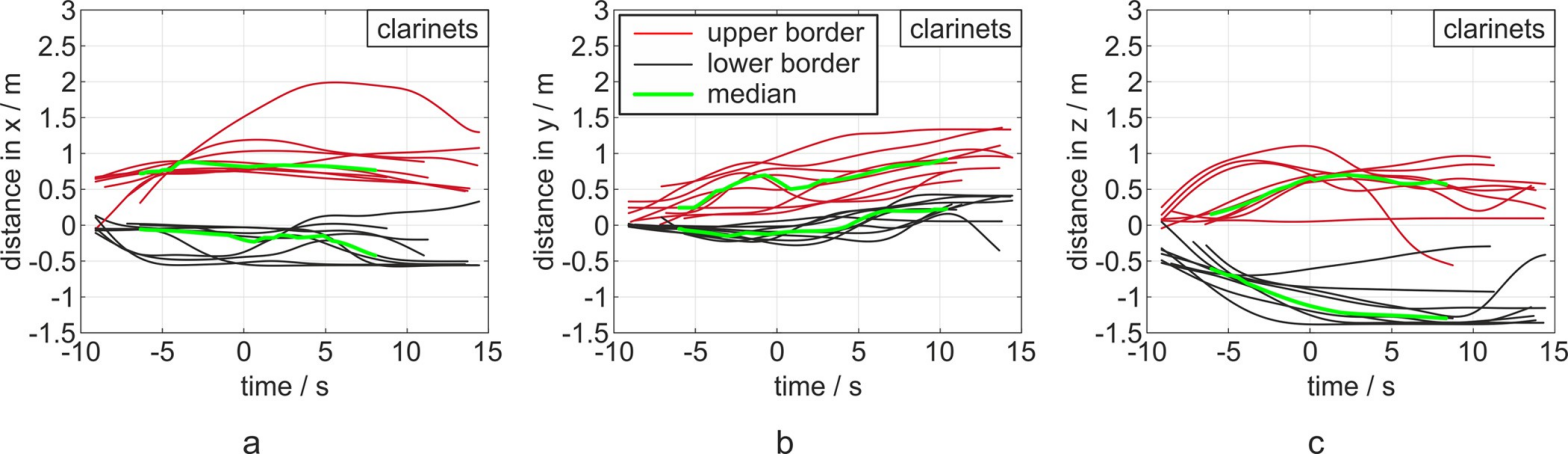
Diagrams of distances in x-, y- and z-direction (a, b, c) from left to right for the clarinets. The 0 point in the time-scale refers to the end of the task. Each task is represented by a red (upper border) and a black curve (lower border) indicating the maximum distances of the cloud in the respective directions. The median devolution in both directions is shown in green. The corresponding diagrams with color-coded notation of the three tasks are displayed in the S3 Fig.

The experiments were performed under controlled conditions, in order to decrease external influences, such as convectional flow. For this reason, spotlights and cameras were positioned in larger distance, the experiment was performed about 5 m to the next wall, the fans of the spotlights were covered, and the room fan was turned off. However, there was a slight convectional flow mainly directed in positive y-direction. Furthermore, there were single subjects who produced an unexpected large distance during a task, caused by additional convection in the x- and z-direction. The most probable reason for the occurrence of the convection flows is the movements of the subjects just before starting the task. The musicians inhaled the aerosols at the right rear end of the platform with the face heading backwards. After the inhalation, the subjects rotated counterclockwise and stepped at least three steps forward to the musician’s mark. This motion potentially induced a combined rotative and translatory flow which resulted in a predominate flow in +y-direction, Figs 4–6B).

After ejection, the aerosol cloud remained in the air, moved and spread in the room driven by its inertia, decelerated and finally dissipated. Three seconds after finishing the tasks the aerosol clouds reached only a median distance of .2 m to the front. Thus, the main distance was reached by the initial ejection with a large impulse. However, it could be assumed that aerosol clouds that accumulate over time constitute a high risk for virus transmission [22] if the sub-micron particles are not removed from the air. Hence, beside the definition of safety distances, the aeration rate of rehearsal rooms and concert halls play a critical role for lowering the transmission risk.

While Abraham et al. [10] showed that the human thermal plume plays an important role under their orchestral hall setting, the impact of the thermal effects of the musicians during playing wind instruments in this study (plume and warmer exhalation flow) on aerosol movement is small. Potential reasons are that the expulsion momentum is much larger than the momentum to the thermal plume and the temperature of the vapor is either cooled to environmental temperature during passing through the instrument or due to the high velocity and the fast dispersion. Furthermore, in both cases, the sub-micron particles are transported too far away from the musician to be in the thermal plume.

The presented study used an artificially-added aerosol with a comparable size range of aerosols (250 - 450 nm) for each task. The realistic number of sub-micron particles expelled during playing instruments is, however, much lower and varies among the different instruments. In this respect, it has been found that counting the number of expelled particles with an aerodynamic particle sizer while playing different wind instruments was between 20 and 2400 particles/litre with an average size of 1.9 to 3.1 μm [9]. Here, the air-jet woodwinds (i.e., flutes) produced the lowest concentration, because a high number of particles was deposited near the inlet [9].

## Limitations

The presented study addresses the aerosol dispersion during time of playing for about 6 to 8 s, so the time for measurement was much too short in comparison to the realistic playing dose in rehearsals or concerts. It could be expected that the accumulation of sub-micron particles in a closed room is dependent on the playing dose.

Beside the accumulation of aerosols, it was reported that during phonation differences in breathing patterns, such as increased minute ventilation and increased tidal volume are likely to be relevant to aerosol transmission, which should also be taken into account during playing instruments [23].

Similarly, the outlet flow and aerosol concentration varies with changes in music amplitude, pitch, and note duration, with different relationships observed for each instrument depending on geometry and playing technique [10]. The trumpet for example also showed a strong inverse correlation between aerosol concentration and note duration, indicating more particles are generated when the notes change quickly, so the reason could be that brass musicians use the motion of their tongues to separate notes. The presented study analysed a short part of the main theme of Ludwig van Beethoven‘s 9^th^ symphony, 4^th^ movement. It cannot be excluded that other melodies or languages would show different results. However, Schiller’s text with Beethoven’s melody has the advantage that both the melody and the text are well known to professional musicians, minimizing artefacts due to learning effects. The musicians were instructed not to move during playing. Therefore, no statement can be made about the extent to which the effect of natural movements of playing in an orchestra and interpreting a piece have an influence on the cloud.

The aerosol cloud consisted of the smoke generated from the basic liquid of e-cigarettes. In contrast to the naturally generated and emitted sub-micron particles, the smoke consists of a much larger amount of aerosol particles and is, thus, visible. As the contour of the cloud was determined with a threshold-based segmentation algorithm, the exact dimensions of the cloud rely on the intensity of the illumination of the particles and the accuracy of the light-sensitive sensor in the camera. As a consequence, this visualization technique does not allow the detection of aerosol particles in regions where the cloud vanished due to evaporation and/or dilution. As the visibility of the vapor cloud is a function of the density of aerosol particles, the decrease in distance caused an uncertainty in the exact location of the border. However, during the relevant period of active playing until time point 0, this uncertainty is estimated to be small owing to the high contrast between cloud and no-cloud regions. For this purpose, other measuring techniques based on particle counting as performed by He et al. have to be applied which, however, deliver the particle count and size only at specific point in the room and thus are not applicable to acquire the spatial and temporal dynamics of an aerosol cloud.

The e-cigarette smoke changed some subjects’ self-sensation during playing–presumably due to changes of the resonance properties of the instrument and the gas column. The professionally trained subjects could, nevertheless, compensate for these changes during the experiment, applying their playing technique also under the experimental circumstances. Moreover, the fact that only professional musicians were included could have influenced the air leakage at the mouthpiece in trumpeters and clarinetists compared to less experienced musicians.

The number of subjects was too small to perform comparative statistical analyses. It could be hoped that a larger number of musicians could be included in future studies in order to test the observed differences and some protective materials to limit the aerosol spread.

## Supporting information

S1 Fig Diagrams of distances in x-, y- and z-direction (a, b, c) from left to right for the trumpet. The 0 point in the time-scale refers to the end of the task. Each task is represented by a solid (upper border) and a dotted curve (lower border) indicating the maximum distances of the cloud in the respective directions. The red curves show the aerosol dispersion for playing the melody on a high pith and loud (Mh+), the blue curves for playing the melody on a high pith and soft (Mh-) and the green curves for playing the melody on a low pitch and loud (Ml+).
(PNG)

S2 Fig Diagrams of distances in x-, y- and z-direction (a, b, c) from left to right for the flute. The 0 point in the time-scale refers to the end of the task. Each task is represented by a solid (upper border) and a dotted curve (lower border) indicating the maximum distances of the cloud in the respective directions. The red curves show the aerosol dispersion for playing the melody on a high pith and loud (Mh+), the blue curves for playing the melody on a high pith and soft (Mh-) and the green curves for playing the melody on a low pitch and loud (Ml+).
(PNG)

S3 Fig Diagrams of distances in x-, y- and z-direction (a, b, c) from left to right for the clarinet. The 0 point in the time-scale refers to the end of the task. Each task is represented by a solid (upper border) and a dotted curve (lower border) indicating the maximum distances of the cloud in the respective directions. The red curves show the aerosol dispersion for playing the melody on a high pith and loud (Mh+), the blue curves for playing the melody on a high pith and soft (Mh-) and the green curves for playing the melody on a low pitch and loud (Ml+).
(PNG)
