# Supplementary figures and images for: Impulse dispersion of aerosols during playing wind instruments
Source: PLoS One. 2022 Mar 3;17(3):e0262994. doi: 10.1371/journal.pone.0262994 (PMC8893631; doi:10.1371/journal.pone.0262994)

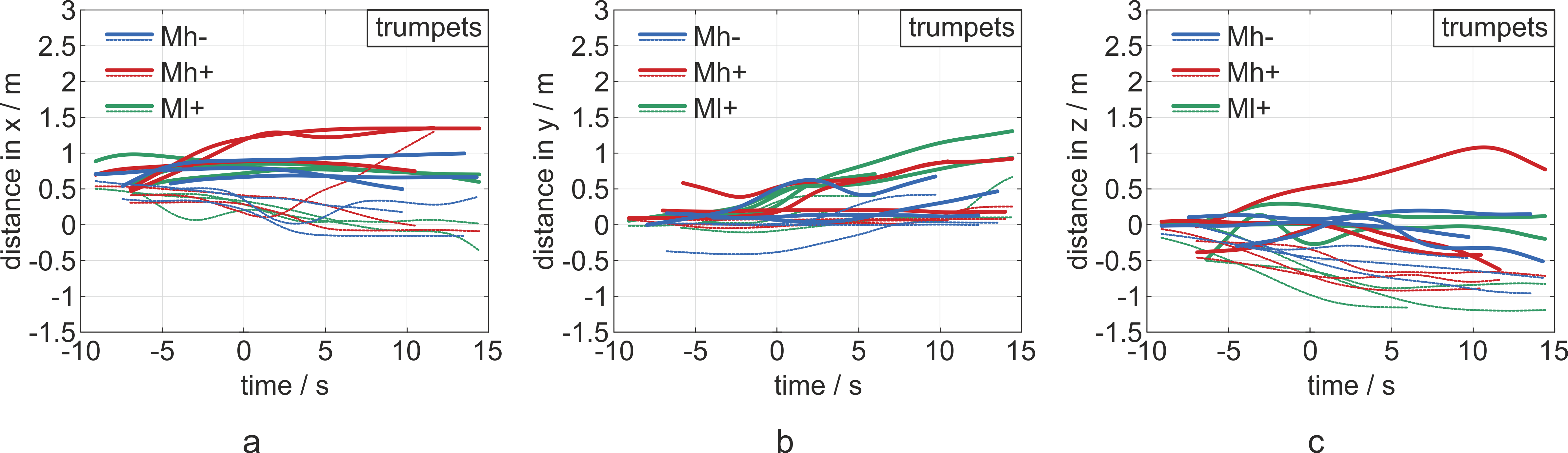

Supplement: S1 Fig — Diagrams of distances in x-, y- and z-direction (a, b, c) from left to right for the trumpet. The 0 point in the time-scale refers to the end of the task. Each task is represented by a solid (upper border) and a dotted curve (lower border) indicating the maximum distances of the cloud in the respective directions. The red curves show the aerosol dispersion for playing the melody on a high pith and loud (Mh+), the blue curves for playing the melody on a high pith and soft (Mh-) and the green curves for playing the melody on a low pitch and loud (Ml+). (PNG) [file pone.0262994.s001.png]

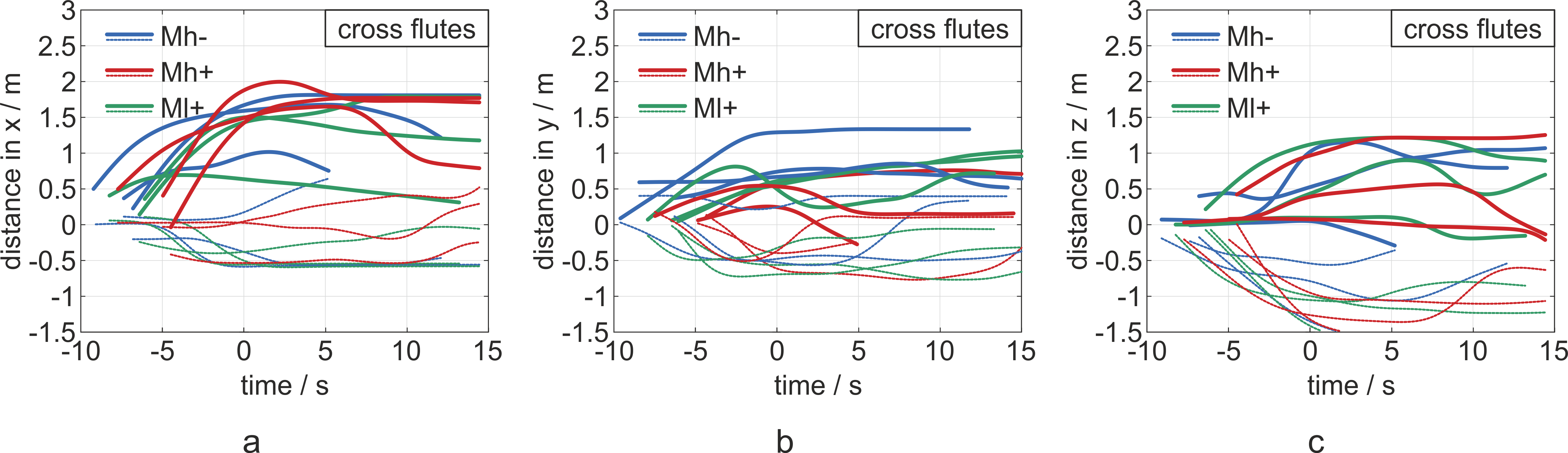

Supplement: S2 Fig — Diagrams of distances in x-, y- and z-direction (a, b, c) from left to right for the flute. The 0 point in the time-scale refers to the end of the task. Each task is represented by a solid (upper border) and a dotted curve (lower border) indicating the maximum distances of the cloud in the respective directions. The red curves show the aerosol dispersion for playing the melody on a high pith and loud (Mh+), the blue curves for playing the melody on a high pith and soft (Mh-) and the green curves for playing the melody on a low pitch and loud (Ml+). (PNG) [file pone.0262994.s002.png]

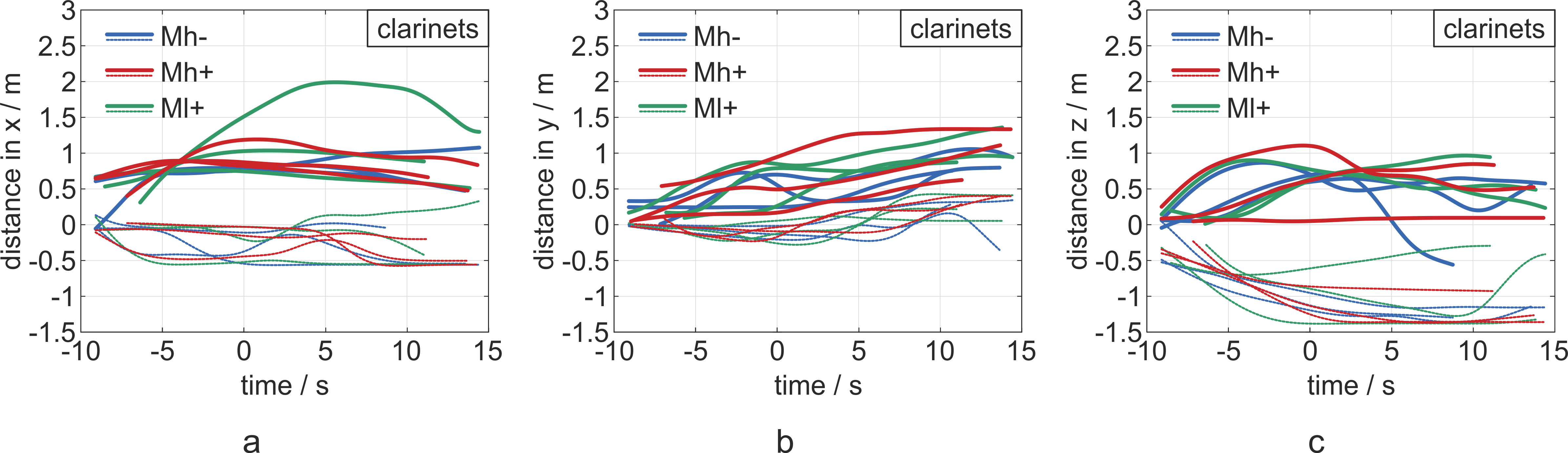

Supplement: S3 Fig — Diagrams of distances in x-, y- and z-direction (a, b, c) from left to right for the clarinet. The 0 point in the time-scale refers to the end of the task. Each task is represented by a solid (upper border) and a dotted curve (lower border) indicating the maximum distances of the cloud in the respective directions. The red curves show the aerosol dispersion for playing the melody on a high pith and loud (Mh+), the blue curves for playing the melody on a high pith and soft (Mh-) and the green curves for playing the melody on a low pitch and loud (Ml+). (PNG) [file pone.0262994.s003.png]
